# Area-level deprivation and preterm birth: results from a national, commercially-insured population

**DOI:** 10.1186/s12889-019-6533-7

**Published:** 2019-02-27

**Authors:** Renee Mehra, Fatma M. Shebl, Shayna D. Cunningham, Urania Magriples, Eric Barrette, Carolina Herrera, Katy B. Kozhimannil, Jeannette R. Ickovics

**Affiliations:** 1 Yale School of Public Health, PO Box 208034, New Haven, CT 06520-8034 USA; 2 Present Address: Medical Practice Evaluation Center, Massachusetts General Hospital, Harvard Medical School, 100 Cambridge Street, Boston, MA 02114 USA; 3 Department of Obstetrics, Gynecology and Reproductive Sciences, Yale School of Medicine, PO Box 208063, New Haven, CT 06520 USA; 4 Health Care Cost Institute, 1100 G Street NW, Suite 600, Washington, DC 20005 USA; 5 Present Address: Medtronic, 950 F Street NW, Suite 500, Washington, DC 20004 USA; 6 Boston University School of Public Health, 715 Albany Street, Boston, MA 02118 USA; 7 Division of Health Policy and Management, University of Minnesota School of Public Health, 420 Delaware Street SE, Minneapolis, MN 55455 USA; 8 Yale-NUS College, 20 College Avenue West #03-401, Singapore, 138529 Singapore

**Keywords:** Commercially-insured, Area-level deprivation, Preterm birth, Mediating pathways

## Abstract

**Background:**

Area-level deprivation is associated with multiple adverse birth outcomes. Few studies have examined the mediating pathways through which area-level deprivation affects these outcomes. The objective of this study was to investigate the association between area-level deprivation and preterm birth, and examine the mediating effects of maternal medical, behavioural, and psychosocial factors.

**Methods:**

We conducted a retrospective cohort study using national, commercial health insurance claims data from 2011, obtained from the Health Care Cost Institute. Area-level deprivation was derived from principal components methods using ZIP code-level data. Multilevel structural equation modeling was used to examine mediating effects.

**Results:**

In total, 138,487 women with a live singleton birth residing in 14,577 ZIP codes throughout the United States were included. Overall, 5.7% of women had a preterm birth. In fully adjusted generalized estimation equation models, compared to women in the lowest quartile of area-level deprivation, odds of preterm birth increased by 9.6% among women in the second highest quartile (odds ratio (OR) 1.096; 95% confidence interval (CI) 1.021, 1.176), by 11.3% in the third highest quartile (OR 1.113; 95% CI 1.035, 1.195), and by 24.9% in the highest quartile (OR 1.249; 95% CI 1.165, 1.339). Hypertension and infection moderately mediated this association.

**Conclusions:**

Even among commercially-insured women, area-level deprivation was associated with increased risk of preterm birth. Similar to individual socioeconomic status, area-level deprivation does not have a threshold effect. Implementation of policies to reduce area-level deprivation, and the screening and treatment of maternal mediators may be associated with a lower risk of preterm birth.

## Background

Ten percent of the four million births in the United States are preterm (birth prior to 37 weeks gestation): a rate relatively intransigent over the past four decades [1]. Preterm birth is a leading cause of infant mortality [2], and preterm infants that survive are susceptible to complications throughout the lifecourse including, respiratory, cardiovascular, and neurological disorders [3]. The consequent social, economic, and human burden is profound.

Both individual- and area-level factors are associated with an increased risk of preterm birth [3]. At the individual level these include sociodemographic characteristics, anthropometrics, infections, and behavioural and psychosocial factors. At the area level these include socioeconomic disadvantage and crime. Although studies have shown an association between area-level socioeconomic disadvantage and poorer birth outcomes, an important limitation of this research is that disadvantage has been measured with numerous indicators, thus making comparisons difficult [4]. In an attempt to create a standardized index, named the neighbourhood deprivation index, Messer and colleagues used principal components analysis to obtain one neighbourhood deprivation factor from 20 variables across seven sociodemographic domains including, education, employment, housing, occupation, poverty, racial  composition, and residential stability [5].

Likewise, the neighbourhood deprivation index has been shown to be associated with poorer birth outcomes [6–10]. However, these studies were limited in scope and scale by being conducted in a small number of geographic regions, and by using vital statistics data which do not contain reliable information or information on potentially important covariates. For example, hypertension, sexually transmitted infections, and mental health conditions are underreported or not included on birth certificates [11, 12]. These limitations may affect the generalizability or conservativeness of study findings. Moreover, studies testing the mechanisms of these associations have been limited [13–16]. Proposed mediators of the association between area-level socioeconomic disadvantage and adverse birth outcomes include biologic, behavioural, psychosocial, and socioeconomic factors at the individual level, and access to goods and services at the area level [14, 17].

The objectives of this study were to examine the association between area-level deprivation and preterm birth, and to assess maternal mediators of this association in a national sample of women. We hypothesize that increasing levels of area-level deprivation will be associated with increased odds of preterm birth, and maternal factors will mediate this association. Using national, commercial health insurance claims data, this study extends the state-of-the-science in the following ways. First, no studies to date have examined the association between area-level deprivation and preterm birth among those commercially insured, despite the fact that commercial health insurance is the most common source of payment for deliveries in the United States, accounting for almost one-half of deliveries [18]. Other sources of payment include Medicaid (43.0%), self-pay or uninsured (4.1%), and other insurance (i.e., other government-funded sources or charity) (3.8%). Second, use of a large national sample of women enables us to explore heterogeneity in the association between area-level deprivation and adverse birth outcomes by geographic region of residence to inform policy making. And third, identifying mediating pathways may guide the development of interventions to prevent adverse birth outcomes.

## Methods

### Study design

This retrospective cohort study used national, commercial health insurance claims data obtained from the Health Care Cost Institute. A convenience sample of de-identified health care services data are provided by three of the largest health insurers (Aetna, Humana, and UnitedHealthcare) from all 50 states. Health Care Cost Institute data represent a quarter of the population with commercial insurance data [19]. Data are compliant with the Health Insurance Portability and Accountability Act, therefore this study was exempt from review by Institutional Review Boards.

### Participants

The study sample was derived and variables defined using information available in claims data, that is Diagnosis Related Group (DRG) payment codes and International Classification of Diseases, Ninth Revision (ICD-9) procedure codes. Following previous research [20], we restricted analyses to women, ages 18 through 44, with a live birth that occurred in an inpatient hospital setting in 2011. These women were covered by employer-sponsored insurance for 10 months prior to childbirth, had a DRG code indicating a vaginal or cesarean delivery (765, 766, 774, or 775), and continued coverage for at least 6 weeks after delivery. To ensure a reliable analytic sample, we excluded patients with any of the following: delivery cannot be clearly identified in inpatient claims due to multiple admission records; non-continuous enrollment (i.e., missing membership records); multiple locations reported during the observation period (i.e., change in residence referenced by change in ZIP code); or event service dates that cross periods (e.g., antepartum outpatient visit with an initiation date prior to admission and an end date following discharge for the index event); multiple gestation (ICD-9 codes 651.00–651.23, 651.80–65.93, V27.2, V27.5); or medical indications justifying preterm delivery including, placenta previa (ICD-9 code 641.XX), and vasa previa (ICD-9 codes 663.51 and 663.53). Less than 5% of claims were excluded [20].

### Variables

#### Individual-level variables

Birth outcome and maternal factors were obtained from the Health Care Cost Institute. Births were classified as preterm using the ICD-9 code 644.2, which indicates deliveries before 37 completed weeks of gestation. Age (18–24, 25–34, 35–44 years), and geographic location (state of residential address converted to one of nine United States Census Bureau divisions [21]), were considered as possible covariates, since preterm birth risk and the neighbourhood deprivation index vary by these factors [5, 7–9]. Specifically, preterm birth risk is higher at younger (less than 25 years) and older (35 years and older) ages [3]. Preterm birth risk differs by race [1], however data on race were not available. Therefore our findings may be overestimated and should be interpreted with caution. Reliable data were not available for other potential confounders such as body mass index (BMI), however BMI is a more important causal determinant of intrauterine growth restriction rather than preterm birth [22], and BMI is often controlled for in studies on low birth weight rather than preterm birth [7].

Maternal factors such as health, health behaviours, psychosocial stress, and individual socioeconomic resources have been conceptualized as mechanisms linking area-level socioeconomic disadvantage and preterm birth [13, 17, 22]. We considered maternal health (medical complications, infection, prior obstetric history, obstetric and fetal complications), health behaviours (substance use), and psychosocial stress (mental health conditions) as potential mediators. Data were not available to test mediating pathways through other maternal factors such as chronic stressors, social support, and individual socioeconomic resources. ICD-9 codes of potential mediators were based on prior research using health insurance claims data [23], which underwent clinical review by a maternal fetal medicine specialist (UM) (see Appendix for detailed list).

#### Area-level variables

We measured exposure to area-level deprivation using the neighbourhood deprivation index, reproducing the methods of Messer and colleagues [5]. This study used ZIP codes of women during pregnancy, which were converted to ZIP Code Tabulation Areas (ZCTA). Unlike many health insurance claims datasets which only include core-based statistical areas (county or counties associated with an urban core [24]), or 3-digit ZIP codes, our data includes 5-digit zip codes. Advantages of using ZIP codes include a large enough scale to protect personal privacy [25], and produce reliable estimates, yet a small enough size to effectively allocate health resources [26].

To compare area-level deprivation across varied geographic locations, preliminary principal component analyses were conducted separately for the nine census divisions. Census divisions are groupings of three to eight adjacent states (and the District of Columbia) that have similar socioeconomics, population characteristics, and historical development [21] (see Table 2 footnotes for the list of states in each division). Principal component analyses were conducted using data from all ZCTAs within a census division. ZCTA-level data were obtained from the 2011 five-year estimates from the American Community Survey [27]. An oblique rotation (promax) was used as the derived factors were correlated (> 0.32), however only the first factor was retained as it accounted for the largest proportion of variability.

Variables were considered for inclusion in the index using a priori criteria: (1) variables loaded above 0.25 in any census division; and (2) lower 95% confidence limit of the variable loading was not below the median lower 95% confidence limit factor loading (0.17 in our analyses) [5]. Ninety-two variables loaded above 0.25 across the census divisions. Bootstrap analyses of 500 randomly drawn samples for each division were used to determine the 95% confidence interval of variable loadings. Forty-two of 92 lower 95% confidence limits did not extend below the absolute value of 0.17. Variables were considered for inclusion if they met these criteria in at least one division. Fourteen variables met all criteria (Table 1), and were retained for final principal component analysis, used to obtain final loadings. ZCTA-level data from all divisions were pooled in the final principal components analysis. Area-level deprivation was standardized to have a mean of zero and a standard deviation of one by dividing the index by the square of the eigenvalue. Higher deprivation values indicate higher levels of deprivation.

**Table 1:** Fourteen variables retained for the final principal component analysis for the area-level deprivation index, 2011

Sociodemographic domain	Variable
Education	% males and females with less than a high school education
Employment	% males and females unemployed
Housing	% housing rented % housing crowded % renter or owner costs in excess of 50% of income
Occupation	% males in professional occupations
Poverty	% households in poverty % female headed households with dependent children % households earning under $30,000/year % households on public assistance % households with no car
Racial composition	% residents who were non-Hispanic blacks
Residential stability	% in same residence since 1995 % residents 65 years and above

Urbanization was measured using rural-urban commuting area (RUCA) codes [28]. RUCA codes are useful in public health studies because they may account for access to health care services [29]. ZIP code approximations of RUCA codes were used [30]. Primary RUCA codes of 10 were considered rural, remaining codes were considered non-rural.

### Statistical methods

The final study sample included women with data on area-level deprivation and preterm birth. Data structure for the analysis was hierarchical: women at level one, areas at level two. Comparisons between unadjusted and adjusted generalized estimating equation models were used to assess for confounding. Only maternal factors diagnosed in at least 1% of the sample were considered for adjustment. We fit generalized estimating equation models to examine the association between area-level deprivation and preterm birth, accounting for similarities among women from the same ZCTA. Model one was the unadjusted model, model two was adjusted by covariates, and model three was adjusted by covariates and potential mediators. Interactions were assessed by adding a cross-product to the model. We allowed for non-linearity by categorizing area-level deprivation into quartiles, similar to previous studies [7, 8]. Other variables were used as categorical variables. A sensitivity analysis for unmeasured confounding was computed using the E-value [31]. Mediation was assessed using multilevel structural equation modeling [32]. Significance level of 0.05 was used. All *P* values were two-sided. Principal component analyses and generalized estimating equation models were performed with SAS software (Version 9.4, The SAS Institute Inc., Cary, NC, 2013). Multilevel structural equation modeling was conducted using Mplus (Version 7, Muthén & Muthén, Los Angeles, CA, 2017).

To minimize potential selection bias and endogeneity due to Health Care Cost Institute data not being a random sample, we assessed the effect of controlling for percentage of individuals in a state covered by commercial health insurance whose data was held by the Health Care Cost Institute in 2015, and ZIP code-level urbanization. 

## Results

A total of 138,494 women from 50 states and the District of Columbia met inclusion criteria for the study. Seven women were missing data on area-level deprivation and were excluded. Overall, 5.7% of women had a preterm birth (Table 2). Preterm births were more common among the youngest and oldest women, and among those residing in the Southeast. Preterm births were less common among women residing along the West and Northeastern coasts. A higher proportion of women with medical complications, infections, poor obstetric history, obstetric and fetal complications, substance use, and mental health conditions had a preterm birth compared to women without these risk factors. Preterm births were more common among women who had a cesarean section compared to a vaginal delivery.

**Table 2:** Characteristics of commercially-insured women by preterm birth status, United States, 2011

Characteristic		Total *N* 138,487	Preterm birth *N* (%) 7870 (5.7)	*P* value^a^
Age at delivery (years)				< 0.001
18–24		16,584	1014 (6.1)	
25–34		92,117	5001 (5.4)	
35–44		29,786	1855 (6.2)	
Census division^b^				< 0.001
New England		4563	233 (5.1)	
Middle Atlantic		18,565	1000 (5.4)	
East North Central		22,524	1240 (5.5)	
West North Central		10,291	561 (5.5)	
South Atlantic		30,527	1876 (6.1)	
East South Central		7615	473 (6.2)	
West South Central		21,985	1316 (6.0)	
Mountain		11,189	616 (5.5)	
Pacific		11,228	555 (4.9)	
Medical factors				
Maternal medical complication				
Hypertension	Present	14,511	1500 (10.3)	< 0.001
	Absent	123,976	6370 (5.1)	
Gestational diabetes	Present	10,639	754 (7.1)	< 0.001
	Absent	127,848	7116 (5.6)	
Pre-existing diabetes	Present	1753	240 (13.7)	< 0.001
	Absent	136,734	7630 (5.6)	
Anaemia	Present	3531	230 (6.5)	< 0.001
	Absent	134,956	7640 (5.7)	
Infection				
Vaginal, sexually transmitted, and systemic infections	Present	38,485	2672 (6.9)	< 0.001
	Absent	100,002	5198 (5.2)	
Prior obstetric history				
Incompetent cervix	Present	2885	479 (16.6)	< 0.001
	Absent	135,602	7391 (5.5)	
Prior preterm birth	Present	2674	576 (21.5)	< 0.001
	Absent	135,813	7294 (5.4)	
Poor obstetric history	Present	5131	513 (10.0)	< 0.001
	Absent	133,356	7357 (5.5)	
Obstetric complication				
Premature rupture of membranes	Present	1717	178 (10.4)	< 0.001
	Absent	136,770	7692 (5.6)	
Fetal complication				
Polyhydramnios	Present	3417	279 (8.2)	< 0.001
	Absent	135,070	7591 (5.6)	
Mode of delivery				< 0.001
Cesarean section		49,495	3371 (6.8)	
Vaginal		88,992	4499 (5.1)	
Behavioural factors				
Substance use (drug, alcohol, and tobacco use)	Present	2153	190 (8.8)	< 0.001
	Absent	136,334	7680 (5.6)	
Psychosocial factors				
Mental health condition (depression, anxiety, and other mental disorders)	Present	7394	555 (7.5)	< 0.001
	Absent	131,093	7315 (5.6)	

^a^*P* values of bivariate associations between characteristics and preterm birth status obtained from generalized estimating equation models

^b^ The United States census divisions are composed of the following states: New England (Connecticut, Maine, Massachusetts, New Hampshire, Rhode Island, Vermont); Middle Atlantic (New Jersey, New York, Pennsylvania); East North Central (Illinois, Indiana, Michigan, Ohio, Wisconsin); West North Central (Iowa, Kansas, Minnesota, Missouri, Nebraska, North Dakota, South Dakota); South Atlantic (Delaware, District of Columbia, Florida, Georgia, Maryland, North Carolina, South Carolina, Virginia, West Virginia); East South Central (Alabama, Kentucky, Mississippi, Tennessee); West South Central (Arkansas, Louisiana, Oklahoma, Texas); Mountain (Arizona, Colorado, Idaho, Montana, Nevada, New Mexico, Wyoming, Utah); and Pacific (Alaska, California, Hawaii, Oregon, Washington)

**Fig. 1:**
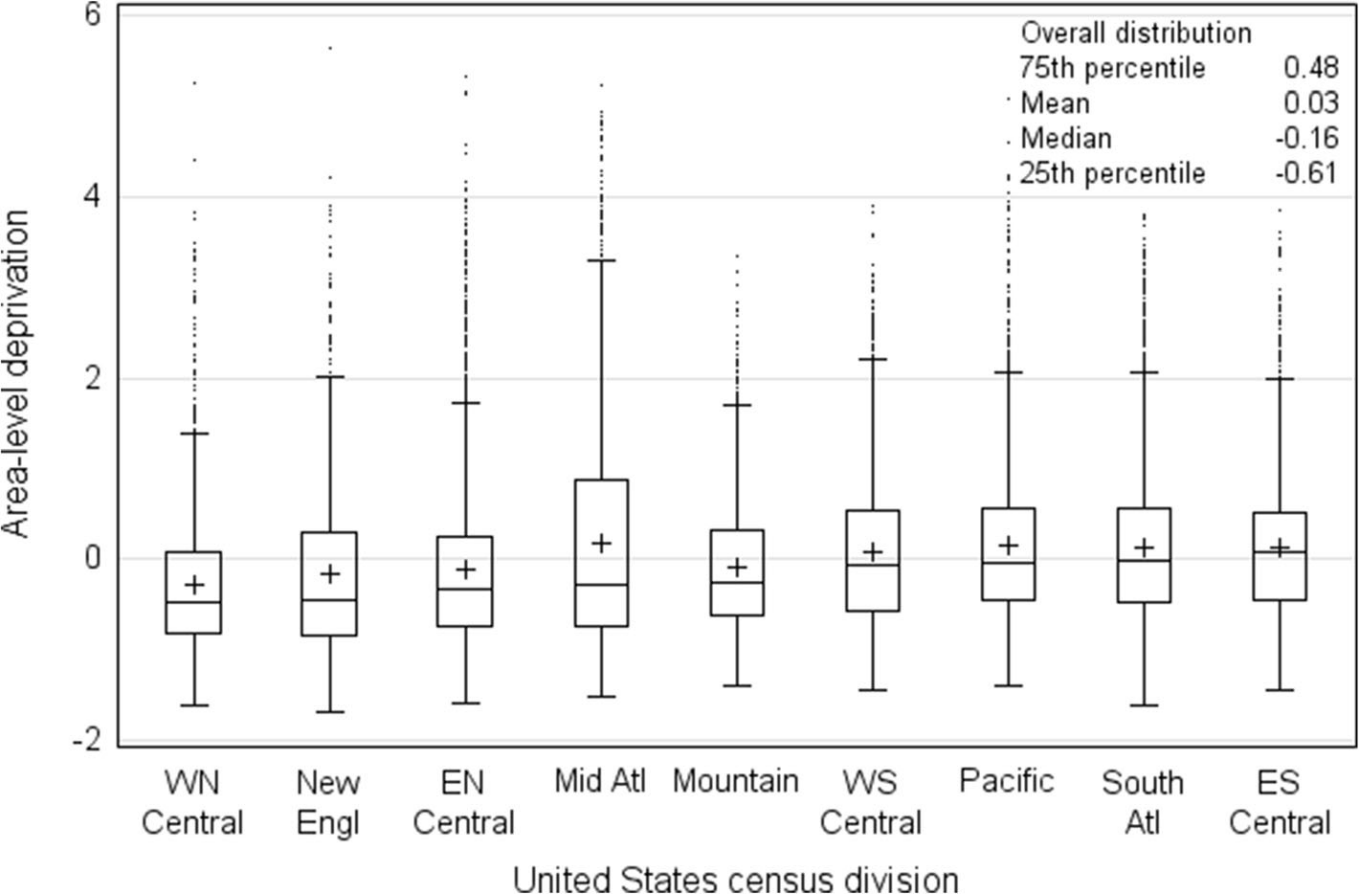
Distribution of area-level deprivation index by census division. + indicates the mean. Dots indicate outliers, which are 1.5 times the interquartile range above the 75th percentile. WN Central, West North Central (Iowa, Kansas, Minnesota, Missouri, Nebraska, North Dakota, South Dakota); New Engl, New England (Connecticut, Maine, Massachusetts, New Hampshire, Rhode Island, Vermont); EN Central, East North Central (Illinois, Indiana, Michigan, Ohio, Wisconsin); Mid Atl, Middle Atlantic (New Jersey, New York, Pennsylvania); Mountain (Arizona, Colorado, Idaho, Montana, Nevada, New Mexico, Wyoming, Utah); WS Central, West South Central (Arkansas, Louisiana, Oklahoma, Texas); Pacific (Alaska, California, Hawaii, Oregon, Washington); South Atl, South Atlantic (Delaware, District of Columbia, Florida, Georgia, Maryland, North Carolina, South Carolina, Virginia, West Virginia); ES Central, East South Central (Alabama, Kentucky, Mississippi, Tennessee)

In final analyses, 138,487 women in 14,577 areas were included. The standardized area-level deprivation index ranged from − 1.67 to 5.63. Distribution of area-level deprivation was fairly homogeneous across census divisions (Fig. 1). Overall, cut-off values were − 0.61, − 0.16 and 0.48 for the lowest (least deprived) to highest (most deprived) quartiles. Overall, the mean was 0.03, skewness was 1.3, and kurtosis was 2.3. By census division, quartile cut-off values were higher in the East South Central, South Atlantic, Pacific, and West South Central divisions. A high proportion of women in the southern, western, and Middle Atlantic census divisions resided in the most deprived areas (Appendix). Area-level deprivation was generally higher in central urban and rural areas (data not shown).

Results of generalized estimating equation models are presented in Table 3. Each increasing quartile of area-level deprivation was associated with population-averaged higher odds of preterm birth. Compared to women in the lowest quartile of area-level deprivation, women in the highest quartile of area-level deprivation had an increased odds of preterm birth in unadjusted (OR 1.33; 95% CI 1.24, 1.42), and fully adjusted analyses (OR 1.25; 95% CI 1.17, 1.34). However, the strength of associations may be overestimated due to our inability to account for race. In sensitivity analyses, the E-value for the highest compared to the lowest quartile of area-level deprivation was 1.807 (or 1.603 for the lower confidence limit). When area-level deprivation was modeled as a continuous variable, the *P* value for trend was < 0.001. State-level coverage of commercial health insurance at the Health Care Cost Institute and urbanization were not included in model three as these variables were not significant predictors. Analyses of effect modification of census division and urbanization were non-significant.

**Table 3:** Unadjusted and adjusted results for preterm birth among commercially-insured women, United States, 2011

Variable	Model 1 (unadjusted)			Model 2 (minimally adjusted)^a^			Model 3 (fully adjusted)^b^		
	OR	95% CI	*P* value	OR	95% CI	*P* value	OR	95% CI	*P* value
Area-level deprivation									
Quartile 1 (least deprived)	1.0			1.0			1.0		
Quartile 2	1.112	1.037,1.193	0.003	1.115	1.039,1.196	0.003	1.096	1.021, 1.176	0.011
Quartile 3	1.152	1.074,1.235	< 0.001	1.143	1.064,1.228	< 0.001	1.113	1.035, 1.195	0.004
Quartile 4 (most deprived)	1.328	1.241,1.421	< 0.001	1.322	1.233,1.417	< 0.001	1.249	1.165, 1.339	< 0.001

^a^ Generalized estimating equation model results controlling for age at delivery and census division

^b^ Generalized estimating equation model results controlling for age at delivery, census division, hypertension, gestational diabetes, pre-existing diabetes, infection, incompetent cervix, poor obstetric history, premature rupture of membranes, mode of delivery, substance use, and mental health conditions

*OR* odds ratio, *CI* confidence interval

**Fig. 2:**
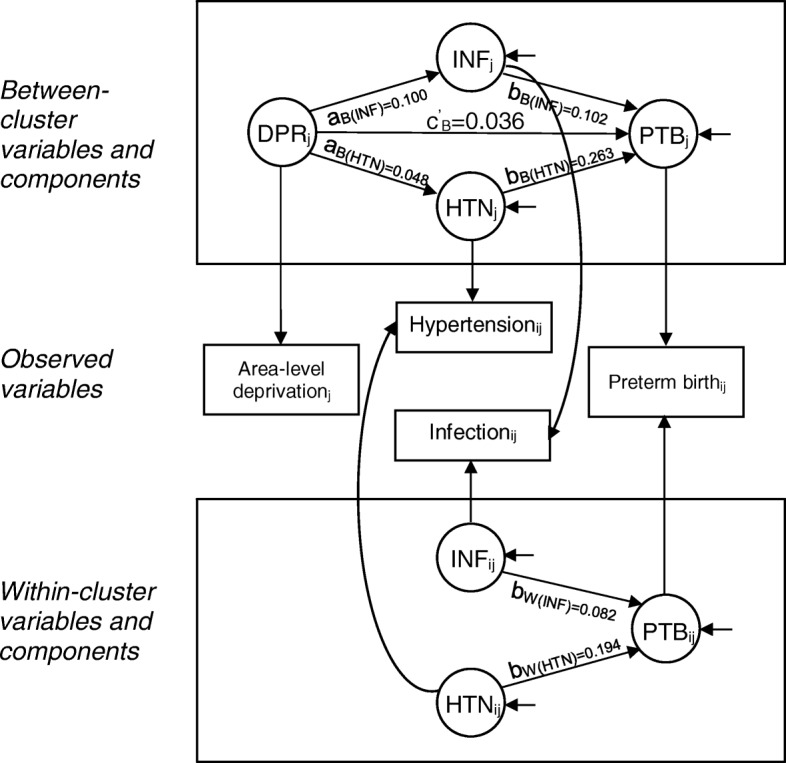
Final structural equation model of area-level deprivation in relation to maternal factors and preterm birth. Note: Parameter estimates are nonstandardized (all *P* values were significant). Rectangular shapes indicate observed variables, circular shapes indicate latent variables. Paths marked with a W subscript denote within-cluster paths, and paths marked with a B subscript denote between-cluster paths. Variables marked with a j subscript are observed at the area level, variables marked with an ij subscript are observed at the individual level within the area level. Arrows from between-cluster variables or within-cluster variables to observed variables indicate the decomposition of observed effects into between and within-cluster effects, respectively. Only the between indirect effect exists because the exposure, mediators, and outcome have between-cluster variation, whereas only the mediators and outcome (but not the exposure) have within-cluster variation. Short arrows entering endogenous variables indicate errors. DPR: area-level deprivation; HTN: hypertension; INF: infection; PTB: preterm birth

The direct association between area-level deprivation (continuous) and preterm birth was confirmed in the multilevel structural equation model (b = 0.036; *P* = 0.001). There was a significant indirect effect of area-level deprivation on preterm birth through hypertension (b = 0.013; *P* = 0.001) and infection (b = 0.010; *P* = 0.006) (Fig. 2 and Appendix). We did not find significant indirect effects through diabetes, substance use, mental health conditions, or other factors. Of the total effect of area-level deprivation on preterm birth, 39% was mediated (i.e., all indirect effects/all effects or 0.023/0.059). Model fit statistics indicated a reasonable fit of the model: root mean square error of approximation (RMSEA) was low (0.025), comparative fit index (CFI) was adequate (0.86), and standardized root mean square residual (SRMR) for within (0.03) and between (0.04) were low.

## Discussion

Area-level deprivation is associated with an increased risk of morbidity and mortality in adulthood [33, 34]. Importantly during pregnancy, when health trajectories are often set for life, area-level deprivation is associated with an increased risk of adverse birth outcomes [35]. In this study, we observed that even among women with commercial health insurance, those that are potentially the most economically advantaged, higher area-level deprivation was associated with an increased risk of preterm birth.

Preterm birth prevalence in this population of commercially-insured women was 5.7%, almost half the national preterm birth rate in 2011 (11.7%) [1]. This was consistent with other studies showing that women with commercial health insurance were less likely to have a preterm birth than women with Medicaid [3]. The range of area-level deprivation index values in this national study were wider (− 1.67 to 5.63) than that found in eight geographic areas in the United States (− 1.85 to 3.72) [5]. However, quartile cut-off values were less extreme in this study (− 0.61, − 0.16 and 0.48 for quartiles one, two and three, respectively) compared to those found by others (− 0.81, − 0.173, and 0.76 for quartiles one, two and three, respectively) [5].

Among commercially-insured women, we observed an increasing trend in the odds of preterm birth as area-level deprivation increased. Note that our findings should be interpreted with caution due to our inability to account for race. Similar to studies examining the association between individual socioeconomic status and health [36], a threshold effect for area-level deprivation was not observed. On average, there was a 25% increase in the odds of preterm birth among women residing in the highest compared to the lowest quartile of area-level deprivation. This estimate may be conservative as we controlled for potential mediators. The effect size of area-level deprivation on preterm birth in our study was similar in magnitude to other studies [7, 8]. Furthermore, in a meta-analysis of seven studies on neighbourhood deprivation measured as a single measure or an index, odds ratios were significantly increased for preterm birth in the most compared to the least deprived neighbourhood quintile (OR 1.23) [35].

Our research adds to these findings by assessing if the association between area-level deprivation and preterm birth is mediated by maternal factors. We found that only hypertension and infection moderately mediated this association. Meng and colleagues showed that approximately 25% of the association between area-level socioeconomic disadvantage and preterm birth and low birthweight was mediated by individual-level factors, with socioeconomic status-related support having the highest level of mediation followed by biologic, behavioural, and psychosocial factors [13]. Schempf and colleagues found that psychosocial mediators reduced the effect of neighbourhood risk on birthweight by 12%, and behavioural mediators reduced the effect by an additional 30% to the point where neighbourhood risk was no longer statistically significant [16]. Meanwhile, area-level factors such as the density of retail outlets for alcohol, tobacco, and unhealthy and healthy foods were unlikely to mediate the association between area-level socioeconomic disadvantage and adverse birth outcomes [14]. However, area-level socioeconomic disadvantage may be associated with other adverse area-level conditions such as inadequate housing and transportation, and violence, which may influence pregnancy through psychological stress [37].

### Strengths and limitations

Strengths of this study included a large sample of commercially-insured women from all 50 states and the District of Columbia, and the availability of potential mediators of the association between area-level deprivation and preterm birth. There is limited research on this association among women with commercial health insurance, who account for almost half of all births [18]. The proportion of births covered by commercial health insurance, particularly after the implementation of the Affordable Care Act (ACA), is growing [38, 39]. This study showed that despite access to commercial health insurance coverage, women who resided in areas with higher deprivation were at greater odds of preterm birth.

Limitations of the study include lack of data on important risk factors for preterm birth (e.g., race and individual socioeconomic status), operationalization of area, area and covariate/mediator misclassification, selection bias, and potential inability to generalize to women without commercial health insurance or residential stability, or women whose commercial health insurers are not included in the Health Care Cost Institute data.

Race is an important predictor of preterm birth, with non-Hispanic black women having a 60% higher prevalence of preterm birth compared to non-Hispanic white women [1]. Other studies show that among women with commercial health insurance, 66.1% are non-Hispanic white, 12.2% are non-Hispanic black, 13.3% are Hispanic, and 8.4% are non-Hispanic other race [40]. Our study population may likewise have a high composition of non-Hispanic white women which may in part explain the low prevalence of preterm birth that was observed. Differences by race/ethnicity were not able to be assessed, therefore our findings may be overestimated. In sensitivity analyses, the minimum strength of an association that an unmeasured confounder would need to have, conditional on measured covariates, to explain away the observed association of the highest compared to the lowest quartile of area-level deprivation was 1.807 (or 1.603 for the lower confidence limit). In a previous study examining neighbourhood deprivation, the adjusted relative risk of preterm birth among non-Hispanic black women compared to non-Hispanic white women was 1.4 (95% CI 1.3–1.6) [41]. This relative risk is less than our E-value which suggests that controlling for race in our analyses would likely not explain away this observed association. However, it may explain away the association for other quartile comparisons. Additionally, results from other studies indicate that adjusting for individual confounders other than race may lead to attenuation of area-level associations [7].

We cannot rule out the possibility that the associations observed in this study could be explained by residual confounding from area-level factors. Although, as indicated above an unmeasured confounder would need to have a relative risk of 1.807 or a lower confidence limit of 1.603 to explain away our observed results. The association between area-level deprivation and preterm birth may be confounded by differences in availability and quality of health care services within an area, which was beyond the scope of the data available. Insurance coverage does not indicate equal access and use of medical care [42]. Furthermore, racial differences in adverse birth outcomes are related to the quality of care at the site of delivery [43].

Areas were operationalized using ZIP codes. ZIP codes are established by the United States Postal Service as mail routes for efficient mail delivery. ZIP codes have a large population size with an average of 30,000 residents and may be socioeconomically heterogeneous [44]. In contrast, census tracts, a common operationalization of neighbourhoods used in public health research, are relatively socioeconomically homogenous statistical subdivisions of counties derived by the United States Census Bureau that have an average of 4000 residents and approximately 1000 to 3000 housing units [21]. Krieger and colleagues compared the associations between socioeconomic measures at the census block group (aggregated census blocks (subdivisions of census tracts) that have approximately 250 to 550 housing units [21]), census tract, and ZIP code level, and birthweight [45]. Measures at the census block group and census tract level showed similar associations, while ZIP-code level measures detected smaller associations. These effect sizes were similar to the effect sizes observed in our study (OR range from 1.27 to 2.17). Therefore, the magnitude of effect size using ZIP code-level data may be smaller, nonetheless significant associations still were detected. Misclassification of areas may arise as ZCTAs (aggregated census blocks that approximate ZIP Codes [46]) and ZIP codes sharing the same code may not cover exactly the same geographic area [44].

Maternal factors and outcomes were obtained from commercial health insurance claims data and their accuracy is dependent on institutional and individual coding. The positive predictor value of chronic conditions and birth outcomes for administrative health plans range from 87 to 95% [47]. Non-differential misclassification of exposures and outcomes would bias our results towards the null, therefore our observed associations could be conservative estimates.

Our study included women with commercial health insurance and residential stability. While the majority of women (53%) in the United States have stable commercial health insurance coverage during pregnancy, women who are less likely to have stable commercial health insurance are younger, non-Hispanic white, have lower socioeconomic status, and initiate prenatal care late [48]. Many of these same factors are associated with residential mobility during pregnancy [49], and preterm birth [3]. Selection bias may have led to an underselection of women with low socioeconomic status who may have had a higher risk of preterm birth. Individual-level socioeconomic status data were not available in this study, thus the direction of bias is unknown.

Our results may or may not generalize to the populations of other insurers, as we were unable to determine how individuals included in the Health Care Cost Institute data compare to individuals covered by other insurers. However, our results are internally valid. Whether the results of this study generalize to all pregnant women, particularly after the implementation of the ACA, is unknown. However, studies that take insurance status into account, still find an association between area-level deprivation and adverse birth outcomes [6].

### Implications for practice and/or policy

We found that area-level deprivation was associated with increased risk of preterm birth among commercially insured women and medical factors mediated this association. The implementation of the ACA was associated with a decrease in being uninsured, and an increase in being commercially insured and receiving timely prenatal care among young women [50]. Thus, efforts to repeal the ACA may result in poorer access to prenatal care and poorer management of medical factors that mediate the association between area-level deprivation and preterm birth, particularly among young women who are at increased risk of preterm birth [3].

Our findings also suggest potential avenues for policy solutions, such as implementing policies to reduce area-level deprivation, and enabling insurers, employers, and health care providers to work towards designing care paths to address medical mediating factors, particularly hypertension and infection.

## Conclusions

Our study showed that in a national sample of commercially-insured women there was notable variation in the area-level deprivation index. Even among this commercially-insured population, there were area-level socioeconomic differences that were related to the health and well-being of mothers and their infants. To reduce the risk of adverse birth outcomes such as preterm birth, it is important to identify both modifiable risk factors and mediators that are potential targets for intervention. Future research should continue to test for mediating pathways between area-level deprivation and preterm birth to determine if hypertension and infection are consistently found to mediate this association, or if there are other important mediating factors. Additional mediating factors to explore include maternal factors such as stress, and area-level conditions such as crime or material and social resources. Longitudinal study designs will enable researchers to consider causal inferences on the effects of area-level factors on preterm birth and other adverse maternal and child health outcomes.

## Appendix

**Table 4:** International Classification of Disease and Diagnosis Related Group payment codes used to identify maternal health conditions

Condition	ICD-9/ DRG code	Definition
Medical factors		
Maternal medical complication		
Hypertension (including preeclampsia and eclampsia)	401.XX	Essential hypertension
	402.XX	Hypertensive heart disease
	403.XX	Hypertensive kidney disease
	404.XX	Hypertensive heart and kidney disease
	405.XX	Secondary hypertension
	642.XX	Hypertension complicating pregnancy childbirth and the puerperium
Gestational diabetes	648.00	Diabetes mellitus complicating pregnancy childbirth or the puerperium
	648.01	Diabetes mellitus of mother with delivery
	648.02	Diabetes mellitus of mother with delivery with postpartum complication
	648.03	Antepartum diabetes mellitus
	648.04	Postpartum diabetes mellitus
	648.8	Abnormal glucose tolerance of mother complicating pregnancy childbirth or the puerperium
	648.80	Abnormal glucose tolerance of mother complicating pregnancy childbirth or the puerperium unspecified as to episode of care
	648.81	Abnormal glucose tolerance of mother with delivery
	648.82	Abnormal glucose tolerance of mother with delivery with postpartum complication
	648.83	Abnormal glucose tolerance of mother antepartum
	648.84	Abnormal glucose tolerance of mother postpartum
Pre-existing diabetes	250.XX	Diabetes mellitus
Obesity	649.1X	Obesity complicating pregnancy, childbirth, or the puerperium
	649.2X	Bariatric surgery status complicating pregnancy, childbirth, or the puerperium
	278.XX	Overweight, obesity and other hyperalimentation
	V85.3X	Adult body mass index ≥30.0 and ≤40.9
	V85.4X	Adult body mass index ≥40.0
Anemia	280	Iron deficiency anemia
	281	Nutritional deficiency anemia
	285.2	Anemia in chronic disease and neoplastic disease
	285.9	Anemia unspecified
	648.2X	Anemia of mother
Cholestasis	646.7X	Liver and biliary tract disorders in pregnancy, antepartum condition or complication
Periodontal disease	523.XX	Gingival and periodontal diseases
IVF	V2385	Pregnancy resulting from assisted reproductive technology
Infection		
Bacteriuria	646.5X	Asymptomatic bacteriuria in pregnancy
	791.9	Bacteriuria
Systemic infection	486	Pneumonia organism unspecified
	540.X	Acute appendicitis
	590.XX	Pyelonephritis or infection of kidney unspecified
Other infections including genital tract infections and STIs	041.0X	Streptococcus infection
	041.5	Hemophilus influenzae (H. influenzae) infection in conditions classified elsewhere and of unspecified site
	041.8X	Other specified bacterial infections
	042.XX	Human immunodeficiency virus (HIV)
	052.XX	Chickenpox
	053.XX	Herpes zoster
	054.XX	Herpes simplex
	056.XX	Rubella
	070	Viral hepatitis
	070.0	Viral hepatitis a with hepatic coma
	070.1	Viral hepatitis a without hepatic coma
	070.2	Viral hepatitis b with hepatic coma
	070.20	Viral hepatitis b with hepatic coma acute or unspecified without hepatitis delta
	070.21	Viral hepatitis b with hepatic coma acute or unspecified with hepatitis delta
	070.3	Viral hepatitis b without mention of hepatic coma
	070.30	Viral hepatitis b without hepatic coma acute or unspecified without hepatitis delta
	070.31	Viral hepatitis b without hepatic coma acute or unspecified with hepatitis delta
	075.XX	Infectious mononucleosis
	078.5	Cytomegaloviral disease
	079.53	Human immunodeficiency virus type 2 [HIV-2]
	079.83	Parvovirus b19
	090.XX	Congenital syphilis
	091.XX	Syphilis
	092.XX	Early syphilis, latent
	093.XX	Cardiovascular syphilis
	094.XX	Neurosyphilis
	095.XX	Other forms of syphilis with symptoms
	096.XX	Late syphilis, latent
	097.XX	Other and unspecified late syphilis
	098.XX	Gonococcal infection
	099.XX	Venereal disease due to chlamydia
	130.XX	Toxoplasmosis
	131.XX	Trichomoniasis
	599.0	Urinary tract infection site not specified
	616.1X	Bacterial vaginosis
	646.6X	Infections of genitourinary tract
	647.60	Other viral diseases of mother complicating pregnancy childbirth or the puerperium unspecified as to episode of care
	647.61	Other viral diseases of mother with delivery
	647.62	Other viral diseases of mother with delivery with postpartum complication
	647.63	Other antepartum viral diseases
	647.64	Other postpartum viral diseases
	771.1	Congenital cytomegalovirus infection
	795.71	Nonspecific serologic evidence of human immunodeficiency virus (HIV)
	V08	Asymptomatic human immunodeficiency virus (HIV) infection status
Prior obstetric history		
Cervical incompetence	654.5x	Cervical incompetence
Previous preterm labor	V23.41	Pregnancy with history of pre-term labor
Poor obstetric history	V23.49 V23.5	Pregnancy with other poor obstetric history Supervision of high-risk pregnancy with other poor obstetric history
Obstetric complication		
Hemorrhage	656.00	Fetal-maternal hemorrhage unspecified as to episode of care in pregnancy
	656.01	Fetal-maternal hemorrhage with delivery
	656.03	Fetal-maternal hemorrhage antepartum condition or complication
Premature rupture of membranes	658.1X	Premature rupture of membranes
	658.20	Delayed delivery after spontaneous or unspecified rupture of membranes unspecified as to episode of care
	658.21	Delayed delivery after spontaneous or unspecified rupture of membranes delivered
	658.23	Delayed delivery after spontaneous or unspecified rupture of membranes antepartum
Fetal complication		
Polyhydramnios	657.XX	Polyhydramnios
Fetal growth	656.50	Poor fetal growth affecting management of mother unspecified as to episode of care
	656.51	Poor fetal growth affecting management of mother delivered
	656.53	Poor fetal growth affecting management of mother and antepartum condition or complication
Mode of delivery		
Cesarean section	DRG765	Cesarean section w cc/mc
	DRG766	Cesarean section w/o cc/mc
Vaginal	DRG774	Vaginal delivery w complicating diagnoses
	DRG775	Vaginal delivery w/o complicating diagnoses
Behavioural factors		
Substance use (drugs, alcohol, and tobacco)	303.XX	Acute alcoholic intoxication or other and unspecified alcohol dependence
	304.XX	Drug dependence
	305.XX	Nondependent abuse of drugs
	648.33	Antepartum drug dependence
	649.0X	Tobacco use disorder complicating pregnancy, childbirth, or the puerperium
Psychosocial factors		
Mental health condition	290.1X	Presenile dementia
	290.4X	Vascular dementia
	291.XX	Alcohol withdrawal and alcohol-induced mental disorders
	292.XX	Drug withdrawal and drug-induced mental disorders
	293.XX	Delirium and psychotic, mood, anxiety and transient mental disorders
	294.XX	Amnestic disorder, dementia, and other persistent mental disorders
	295.XX	Schizophrenia
	296.XX	Bipolar, manic affective, major depressive and episodic mood disorders
	297.X	Paranoid state, delusional, paraphrenia and shared psychotic disorders
	298.X	Depressive type psychosis, excitative type psychosis, reactive confusion, acute paranoid reaction, psychogenic paranoid psychosis, other and unspecified reactive psychosis and unspecified psychosis
	299.XX	Autistic disorder, childhood disintegrative disorder, other or unspecified pervasive developmental disorder
	300.XX	Anxiety state unspecified, panic disorder without agoraphobia, generalized anxiety disorder, other anxiety states, hysteria unspecified, conversion disorder, dissociative amnesia, dissociative fugue, dissociative identity disorder, dissociative disorder or reaction unspecified, factitious disorder with predominantly psychological signs and symptoms, other and unspecified factitious illness, phobia unspecified, agoraphobia with panic disorder, agoraphobia without panic attacks, social phobia, other isolated or specific phobias, obsessive-compulsive disorders, dysthymic disorder, neurasthenia, depersonalization disorder, hypochondriasis, somatization disorder, undifferentiated somatoform disorder, other somatoform disorders, unspecified nonpsychotic mental disorder
	301.XX	Paranoid personality disorder, affective personality disorder unspecified, chronic hypomanic personality disorder, chronic depressive personality disorder, cyclothymic disorder, schizoid personality disorder unspecified, introverted personality, schizotypal personality disorder, explosive personality disorder, obsessive-compulsive personality disorder, histrionic personality disorder unspecified, chronic factitious illness with physical symptoms, other histrionic personality disorder, dependent personality disorder, antisocial personality disorder, narcissistic personality disorder, avoidant personality disorder, borderline personality disorder, passive-aggressive personality, other personality disorders, unspecified personality disorder
	302.XX	Unspecified psychosexual disorder
	317	Mild intellectual disabilities
	318.X	Moderate, severe and profound intellectual disabilities
	319	Unspecified intellectual disabilities
	648.4X	Mental disorders of mother

*DRG* Diagnosis Related Group, *HIV* human immunodeficiency virus, *ICD-9* International Classification of Diseases, Ninth Revision, *IVF* in vitro fertilization, *STI* sexually transmitted infection

**Table 5:** Distribution of commercially-insured women by quartile of area-level deprivation and census division, United States, 2011

Census division^a^	Area-level deprivation (quartiles) *N* (%)				Total *N* 138,487
	1 (least deprived)	2	3	4 (most deprived)	
New England	1826 (40.0)	1018 (22.3)	809 (17.7)	910 (19.9)	4563
Middle Atlantic	5982 (32.2)	4211 (22.7)	2187 (11.8)	6185 (33.3)	18,565
East North Central	6918 (30.7)	5850 (26.0)	5445 (24.2)	4311 (19.1)	22,524
West North Central	3980 (38.7)	2804 (27.2)	2233 (21.7)	1274 (12.4)	10,291
South Atlantic	5052 (16.5)	7847 (25.7)	8872 (29.1)	8756 (28.7)	30,527
East South Central	1274 (16.7)	1590 (20.9)	2714 (35.6)	2037 (26.7)	7615
West South Central	5152 (23.4)	4680 (21.3)	6159 (28.0)	5994 (27.3)	21,985
Mountain	3016 (27.0)	3084 (27.6)	2907 (26.0)	2182 (19.5)	11,189
Pacific	1401 (12.5)	3316 (29.5)	3429 (30.5)	3082 (27.4)	11,228

^a^ The United States census divisions are composed of the following states: New England (Connecticut, Maine, Massachusetts, New Hampshire, Rhode Island, Vermont); Middle Atlantic (New Jersey, New York, Pennsylvania); East North Central (Illinois, Indiana, Michigan, Ohio, Wisconsin); West North Central (Iowa, Kansas, Minnesota, Missouri, Nebraska, North Dakota, South Dakota); South Atlantic (Delaware, District of Columbia, Florida, Georgia, Maryland, North Carolina, South Carolina, Virginia, West Virginia); East South Central (Alabama, Kentucky, Mississippi, Tennessee); West South Central (Arkansas, Louisiana, Oklahoma, Texas); Mountain (Arizona, Colorado, Idaho, Montana, Nevada, New Mexico, Wyoming, Utah); and Pacific (Alaska, California, Hawaii, Oregon, Washington)

**Table 6:** Multilevel structural equation model parameter estimates for the association between area-level deprivation and preterm birth

	Nonstandardized estimates		Standardized estimates	
	b (SE)	*P* value	β (SE)	*P* value
Area level				
Preterm regressed on				
Hypertension	0.263 (0.077)	0.001	0.400 (0.117)	0.001
Infection	0.102 (0.037)	0.006	0.230 (0.085)	0.007
Deprivation (direct effect)	0.036 (0.008)	<0.001	0.288 (0.076)	<0.001
Hypertension regressed on deprivation	0.048 (0.006)	<0.001	0.249 (0.030)	<0.001
Infection regressed on deprivation	0.100 (0.005)	<0.001	0.351 (0.017)	<0.001
Indirect effects				
Deprivation-hypertension-PTB	0.013 (0.004)	0.001	-	-
Deprivation-infection-PTB	0.010 (0.004)	0.006	-	-
All indirect effects	0.023 (0.006)	<0.001	-	-
All effects	0.059 (0.006)	<0.001	-	-
Individual level				
Preterm birth regressed on				
Hypertension	0.194 (0.009)	<0.001	0.190 (0.009)	<0.001
Infection	0.082 (0.008)	<0.001	0.081 (0.008)	<0.001

## Abbreviations

ACA: Affordable Care Act
BMI: Body mass index
CFI: Comparative fit index
CI: Confidence interval
DPR: Area-level deprivation
DRG: Diagnosis Related Group
EN Central: East North Central
ES Central: East South Central
HTN: Hypertension
ICD-9: International Classification of Diseases, Ninth Revision
INF: Infection
Mid Atl: Middle Atlantic
New Engl: New England
OR: Odds ratio
PTB: Preterm birth
RMSEA: Root mean square error of approximation
RUCA: Rural-urban commuting area
South Atl: South Atlantic
SRMR: Standardized root mean square residual
WN Central: West North Central
WS Central: West South Central
ZCTA: ZIP Code Tabulation Area

## References

[1] Martin JA, Hamilton BE, Osterman MJ, Curtin SC, Matthews TJ (2015). Births: final data for 2013. Natl Vital Stat Rep.

[2] Mathews TJ, MacDorman MF, Thoma ME (2015). Infant mortality statistics from the 2013 period linked birth/infant death data set. Natl Vital Stat Rep..

[3] Institute of Medicine (2007). Preterm birth: causes, consequences, and prevention.

[4] Kim D, Saada A (2013). The social determinants of infant mortality and birth outcomes in Western developed nations: a cross-country systematic review. Int J Environ Res Public Health.

[5] Messer LC, Laraia BA, Kaufman JS, Eyster J, Holzman C, Culhane J (2006). The development of a standardized neighborhood deprivation index. J Urban Health.

[6] Wentz AE, Messer LC, Nguyen T, Boone-Heinonen J (2014). Small and large size for gestational age and neighborhood deprivation measured within increasing proximity to homes. Health Place.

[7] Janevic T, Stein CR, Savitz DA, Kaufman JS, Mason SM, Herring AH (2010). Neighborhood deprivation and adverse birth outcomes among diverse ethnic groups. Ann Epidemiol.

[8] Ma XG, Fleischer NL, Liu JH, Hardin JW, Zhao G, Liese AD (2015). Neighborhood deprivation and preterm birth: an application of propensity score matching. Ann Epidemiol.

[9] O'Campo P, Burke JG, Culhane J, Elo IT, Eyster J, Holzman C (2008). Neighborhood deprivation and preterm birth among non-Hispanic black and white women in eight geographic areas in the United States. Am J Epidemiol.

[10] Elo IT, Culhane JF, Kohler IV, O'Campo P, Burke JG, Messer LC (2009). Neighbourhood deprivation and small-for-gestational-age term births in the United States. Paediatr Perinat Epidemiol.

[11] Lydon-Rochelle MT, Holt VL, Cardenas V, Nelson JC, Easterling TR, Gardella C (2005). The reporting of pre-existing maternal medical conditions and complications of pregnancy on birth certificates and in hospital discharge data. Am J Obstet Gynecol.

[12] Roohan PJ, Josberger RE, Acar J, Dabir P, Feder HM, Gagliano PJ (2003). Validation of birth certificate data in New York State. J Community Health.

[13] Meng G, Thompson ME, Hall GB (2013). Pathways of neighbourhood-level socio-economic determinants of adverse birth outcomes. Int J Health Geogr.

[14] Farley TA, Mason K, Rice J, Habel JD, Scribner R, Cohen DA (2006). The relationship between the neighbourhood environment and adverse birth outcomes. Paediatr Perinat Epidemiol.

[15] Clayborne ZM, Giesbrecht GF, Bell RC, Tomfohr-Madsen LM (2017). Relations between neighbourhood socioeconomic status and birth outcomes are mediated by maternal weight. Soc Sci Med.

[16] Schempf A, Strobino D, O'Campo P (2009). Neighborhood effects on birthweight: an exploration of psychosocial and behavioral pathways in Baltimore, 1995-1996. Soc Sci Med.

[17] Culhane JF, Elo IT (2005). Neighborhood context and reproductive health. Am J Obstet Gynecol.

[18] Martin JA, Hamilton BE, Osterman MJK, Driscoll AK, Drake P (2018). Births: final data for 2017. Natl Vital Stat Rep..

[19] Health Care Cost Institute. Health Care Cost and Utilization Report: 2011. 2012. https://www.healthcostinstitute.org/images/pdfs/2011-HCCI-Annual-Report.pdf. Accessed 16 Jan 2019.

[20] Cunningham SD, Herrera C, Udo IE, Kozhimannil KB, Barrette E, Magriples U (2017). Maternal medical complexity: impact on prenatal health care spending among women at low risk for cesarean section. Womens Health Issues.

[21] Bureau of the Census. Geographic areas reference manual. 1994. https://www.census.gov/geo/reference/garm.html. Accessed 27 Jan 2016.

[22] Kramer MS, Seguin L, Lydon J, Goulet L (2000). Socio-economic disparities in pregnancy outcome: why do the poor fare so poorly?. Paediatr Perinat Epidemiol.

[23] Xu X, Gariepy A, Lundsberg LS, Sheth SS, Pettker CM, Krumholz HM (2015). Wide variation found in hospital facility costs for maternity stays involving low-risk childbirth. Health Aff (Millwood).

[24] United States Census Bureau. Geographic terms and concepts - core based statistical areas and related statistical areas. https://www.census.gov/geo/reference/gtc/gtc_cbsa.html. Accessed 6 Jan 2019.

[25] Jacquez GM (2004). Current practices in the spatial analysis of cancer: flies in the ointment. Int J Health Geogr.

[26] Tian N, Goovaerts P, Zhan FB, Wilson JG (2010). Identification of racial disparities in breast cancer mortality: does scale matter?. Int J Health Geogr.

[27] United States Census Bureau. ACS 2011 (5-year estimates). Prepared by Social Explorer. Accessed 27 Jan 2016.

[28] United States Department of Agriculture. Documentation: 2010 Rural-Urban Commuting Area (RUCA) codes. https://www.ers.usda.gov/data-products/rural-urban-commuting-area-codes/documentation/. Accessed 12 Feb 2018.

[29] Hall SA, Kaufman JS, Ricketts TC (2006). Defining urban and rural areas in U.S. epidemiologic studies. J Urban Health.

[30] Center for Rural Health. Temporary ZIP RUCA 3.10 file access. https://ruralhealth.und.edu/ruca. Accessed 12 Feb 2018.

[31] VanderWeele TJ, Ding P (2017). Sensitivity analysis in observational research: introducing the e-value. Ann Intern Med.

[32] Preacher KJ, Zyphur MJ, Zhang Z (2010). A general multilevel SEM framework for assessing multilevel mediation. Psychol Methods.

[33] White JS, Hamad R, Li X, Basu S, Ohlsson H, Sundquist J (2016). Long-term effects of neighbourhood deprivation on diabetes risk: quasi-experimental evidence from a refugee dispersal policy in Sweden. Lancet Diabetes Endocrinol.

[34] Meijer M, Rohl J, Bloomfield K, Grittner U (2012). Do neighborhoods affect individual mortality? A systematic review and meta-analysis of multilevel studies. Soc Sci Med.

[35] Vos AA, Posthumus AG, Bonsel GJ, Steegers EA, Denktas S (2014). Deprived neighborhoods and adverse perinatal outcome: a systematic review and meta-analysis. Acta Obstet Gynecol Scand.

[36] Marmot M (2015). The health gap: the challenge of an unequal world.

[37] Messer LC, Maxson P, Miranda ML (2013). The urban built environment and associations with women's psychosocial health. J Urban Health.

[38] Akosa Antwi Y, Ma J, Simon K, Carroll A (2016). Dependent coverage under the ACA and Medicaid coverage for childbirth. N Engl J Med.

[39] Daw JR, Sommers BD (2018). Association of the Affordable Care act dependent coverage provision with prenatal care use and birth outcomes. JAMA..

[40] Markus AR, Krohe S, Garro N, Gerstein M, Pellegrini C. Examining the association between Medicaid coverage and preterm births using 2010-2013 National Vital Statistics Birth Data. J Child Poverty. 2016:1–16 10.1080/10796126.2016.1254601.

[41] Schempf AH, Kaufman JS, Messer LC, Mendola P (2011). The neighborhood contribution to black-white perinatal disparities: an example from two North Carolina counties, 1999-2001. Am J Epidemiol.

[42] Adler NE, Stewart J (2010). Health disparities across the lifespan: meaning, methods, and mechanisms. Ann N Y Acad Sci.

[43] Howell EA, Egorova NN, Janevic T, Balbierz A, Zeitlin J, Hebert PL (2017). Severe maternal morbidity among Hispanic women in New York City: investigation of health disparities. Obstet Gynecol.

[44] Krieger N, Waterman P, Chen JT, Soobader MJ, Subramanian SV, Carson R (2002). Zip code caveat: bias due to spatiotemporal mismatches between zip codes and US census-defined geographic areas--the public health disparities geocoding project. Am J Public Health.

[45] Krieger N, Chen JT, Waterman PD, Soobader MJ, Subramanian SV, Carson R (2003). Choosing area based socioeconomic measures to monitor social inequalities in low birth weight and childhood lead poisoning: the public health disparities geocoding project (US). J Epidemiol Community Health.

[46] US Bureau of the Census. ZIP Code™ Tabulation Areas (ZCTAs™). https://www.census.gov/geo/reference/zctas.html. Accessed 4 Jan 2019.

[47] Andrade SE, Scott PE, Davis RL, Li DK, Getahun D, Cheetham TC (2013). Validity of health plan and birth certificate data for pregnancy research. Pharmacoepidemiol Drug Saf.

[48] D'Angelo DV, Le B, O'Neil ME, Williams L, Ahluwalia IB, Harrison LL (2015). Patterns of health insurance coverage around the time of pregnancy among women with live-born infants--pregnancy risk assessment monitoring system, 29 states, 2009. MMWR Surveill Summ.

[49] Bell ML, Belanger K (2012). Review of research on residential mobility during pregnancy: consequences for assessment of prenatal environmental exposures. J Expo Sci Environ Epidemiol.

[50] Li R, Bauman B, D'Angelo DV, Harrison LL, Warner L, Barfield W, Cox S (2019). Affordable care act-dependent insurance coverage and access to care among young adult women with a recent live birth. Med Care.

